# Association of Leisure Activities With Cognitive Impairment and Dementia in Older Adults in Colombia: A SABE-Based Study

**DOI:** 10.3389/fneur.2021.629251

**Published:** 2021-03-01

**Authors:** Alejandra Guerrero Barragán, Diego Lucumí, Brian Lawlor

**Affiliations:** ^1^Global Brain Health Institute, Trinity College Dublin, Dublin, Ireland; ^2^Global Brain Health Institute, University of California, San Francisco, San Francisco, CA, United States; ^3^Escuela de Gobierno, Universidad de los Andes, Bogotá, Colombia; ^4^Unidad de Servicios de Salud Occidente de Kennedy, Servicio de Neurología, Bogotá, Colombia; ^5^Department of Psychiatry, Mercer's Institute for Successful Ageing, St. James's Hospital, Dublin, Ireland

**Keywords:** prevention, cognitive reserve, leisure activities, dementia, cognitive impairment

## Abstract

Observational and interventional studies suggest that participation in leisure activities may help protect against cognitive decline in older people. This study aimed to examine the association between participation in leisure activities and cognitive impairment in older adults in Colombia. Data for this study were derived from the Colombian National Survey of Aging (SABE 2015), a cross-sectional survey with a sample size of 23,694 older adults representing the total population (mean age, 70.8 years; 57.3% females). Cognitive impairment was classified as cognitive impairment without dementia (CIWD) and dementia, according to the revised version of the Folstein Mini-Mental State Examination and the Lawton and Brody functional scale. Leisure activities were evaluated using six items of a questionnaire. Sex-stratified multinomial regression models were used to analyze the association of leisure activities with CIWD and dementia after adjusting for educational attainment, literacy, and other potential confounders. In adjusted models for men, leisure activities in later life were associated with a decreased risk of CIWD (odds ratio [OR], 0.73; 95% confidence interval [CI], 0.68–0.78) and dementia (OR, 0,52; 95% CI, 0.48–0.58). For women, leisure activities in later life were associated with a decreased risk of CIWD (OR, 0.72; 95% CI, 0.66–0.78) and dementia (OR, 0.48; 95% CI, 0.43–0.53). The findings suggest that greater participation in leisure activities in later life may act as a protective factor against CIWD and dementia among older adults in Colombia, independent of educational attainment and literacy.

## Introduction

With a rise in global life expectancy, the prevalence of dementia is rising. A new diagnosis of dementia is reported every 3 s. Currently, the disease affects more than 50 million people globally (1), 50% of those living in middle- and low-income countries. It is expected that this number will rise to 63% in 2030 and 68% in 2050 (2), with the Americas being one of the most affected regions with increasing prevalence and incidence rates of dementia (3).

Several protective factors for dementia have been described in the literature (4). Leisure activities, a term that refers to a range of tasks and activities outside work-related activities is a promising factor for dementia prevention (5). Leisure activities can have a multidimensional profile, manifesting mental, social, and physical involvement (6). These activities are attractive, pleasant, and motivating to the individual and are more likely to be sustained over time (7, 8). Leisure activities usually constitute a relatively large part of daily life post-retirement and may provide mental stimulation, social engagement, and physical activity (6). Systematic reviews have found evidence suggesting that participation in leisure activities might significantly contribute to the prevention of later-life cognitive decline as a risk-preventing factor (5, 9). The most common categories associated with the preventive effect of leisure activities are cognitive, physical, and social activities (5, 10–12). Despite these benefits, there is a lack of standard definitions, measures, and methods for studying the role of leisure activities and their protective effects.

The most common explanatory mechanism for the preventive effect of leisure activities is that of the cognitive reserve theory (5, 13). The cognitive reserve theory suggests that innate intelligence or life experiences such as educational or occupational attainments may supply a reserve in the form of skill sets or repertoires that empower the brain to tolerate atrophies and insults and, as a result, allow some people to cope with progressing dementia better than others and delay symptom onset (14, 15). The cognitive reserve cannot be observed or directly measured, and proxy measures such as education, premorbid intelligence (IQ), linguistic ability, and occupational complexity are often used (14). Several studies have proposed that engagement in leisure activities may result in more functionally efficient or resilient cognitive networks or in recruiting alternate networks, providing a cognitive reserve that prevents or delays the onset of dementia (16–18). In this regard, it is unclear whether a high cognitive reserve delays symptoms onset, but any noticeable delay, for example, 1 or 2 years, would translate into tremendous public health benefits by reducing the prevalence of dementia in the population (14). While characteristics such as education and intelligence are relatively stable from young adulthood, there is increasing interest in the role of leisure activities in building up a cognitive reserve (14).

Although maintaining or building a cognitive reserve is a possible mechanism for dementia prevention (5, 9, 14), culture, poverty, and inequality are key obstacles to, and drivers of, the need for changes concerning the cognitive reserve. Sections of the society that are most deprived of basic needs, like low and middle income countries (LMIC), require these changes and will greatly benefit from them (4). It has been suggested that a low socioeconomic status (SES) is associated with less access to physical (19) and cognitive stimulating activities (20). In Latin America, low levels of education, high rates of brain injury, poor diet, a sedentary lifestyle, and a high risk of cardiovascular diseases are among the main risk factors behind the rapid growth in the number of people with dementia (21); however, there is a high potential for dementia prevention in the region (4, 22). Thus, there is an urgent need for dementia prevention policies in Latin America to reduce the burden and economic costs of dementia in the region (23–25).

In this study, we detail the association between participation in leisure activities and the risk of developing cognitive impairment without dementia (CIWD), or dementia, using the data from the Colombian National Survey of Aging (SABE 2015) (26). There are few papers focusing on cognitive outcomes from Colombian SABE, the effect of education in early life and the probability of cognitive impairment in later life in Colombia has been recently published (27) as well as the role of gait speed in dementia (28) and the mediating effect of physical fitness on cognitive functioning (29). To the best of our knowledge, this is the first study to analyze this topic in Colombia using this dataset.

## Materials and Methods

We conducted a secondary data analysis of a cross-sectional study using data from SABE Colombia 2015. SABE Colombia 2015 is the first study in Colombia representative of the national population of those aged at least 60 years. Individuals for SABE Colombia were selected following a multistage area probability sample design with a total sample size of 23,694 participants from 244 municipalities (urban and rural). Data collection took place between April and September 2015 (26). This study was approved by the Institutional Review Board of Universidad de los Andes, code ID 1114/2019.

The dependent variable was cognitive impairment, as measured in SABE 2015. This survey used the revised version of the Folstein Mini-Mental State Examination (MMSE), a validated international scale translated to Spanish (30). A cut-off point of 12 or less indicated cognitive impairment, while a score of 13 or above was normal (26). For dementia, functional impairment was evaluated using four items of the Lawton and Brody functional scale (31): phone use, transport use, handling medicines, and management of money. The lack of functionality for doing at least two of the four activities was defined as dependence and indication of dementia (32). If functional impairment was not detected, participants were classified as CIWD.

Independent variables were educational attainment, literacy (reading and writing), and participation in leisure activities. Educational attainment was established based on the 11 categories reported by SABE 2015. We classified participants into three groups in accordance with their educational qualifications: those who had an educational qualification lower than completion of primary school, those who had completed primary school, and those who had sought further qualifications after completion of high school. Literacy was reported using a yes/no question. Using an 18-item questionnaire reported by SABE 2015, we constructed a 6-item continuous variable for participation in leisure activities, among which five items were considered cognitively challenging (reading, solving math problems, solving puzzles, tabletop games, attending classes or courses) and one item that included physical activity.

Confounding variables included were age in years, area of residence (urban, rural), marital status (married/with a partner, separated/widower, single), living alone (yes/no), health insurance (subsidized, contribute, no affiliation), antecedent of forced displacement during life (yes/no), country region (residence in one of the six geographic areas of Colombia: Atlantic, Oriental, Orinoquia and Amazonia, Bogotá, Central, Pacific), skin color (light, medium, dark), pension (yes/no), lifetime occupation (manual or dependent worker, boss or independent worker, none), salary (<1 minimum wage, between 1 and 2 minimum wages, more than 2 minimum wages), and physical capital tercile (1–3).

### Inclusion and Exclusion Criteria

The present study included the total SABE survey sample: participants aged 60 years and above, non-institutionalized, capable of communicating with the research team and able to provide written informed consent. If the MMSE was below 13 a proxy interview was developed (26).

Participants with missing data for leisure activities participation were excluded from this study.

### Statistical Analyses

Descriptive analyses of data were conducted using frequencies and percentages for qualitative variables and means and standard deviations for quantitative variables. Unadjusted odds ratio (OR) was calculated using multinomial regression logistic analyses for dependent and independent variables. Multicollinearity was test using variance inflation factor (VIF). Finally, sex-stratified multinomial regression models were used to examine the association of leisure activities with CI and dementia after adjusting for educational attainment, literacy, and other potential confounders. Results are presented as OR with 95% confidence intervals. All analyses were performed using STATA release 16® (StataCorp LP, College Station, USA).

## Results

**Figure 1:**
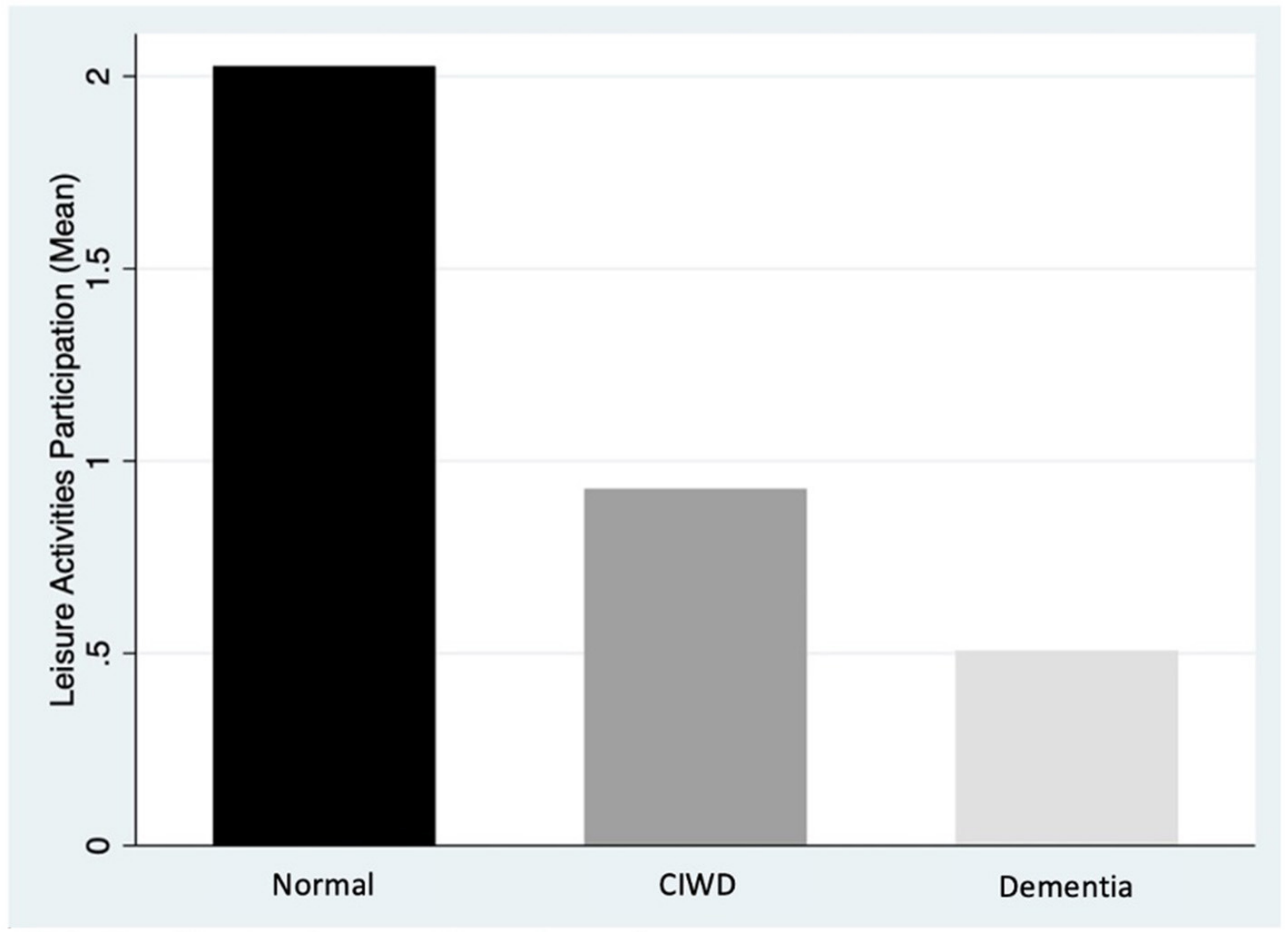
Mean leisure activities participation. CIWD, Cognitive impairment without dementia.

The sample size of the survey was 23,694 participants, 31 participants with missing data for leisure activities were excluded from this study, the final sample size was 23.663 with a mean age of 70.82 years (standard deviation [SD] 8.20); 57.3% were women. Most participants had an educational qualification lower than completion of primary school (62.6%), followed by those who had completed primary school (26.8%) and sought further qualifications after completion of high school (10.5%). Literacy was reported as 78.2 and 77.1% for reading and writing, respectively. The prevalence of CIWD was 8.9 and 10.8% for dementia (Table 1). The mean number of leisure activities participated in was 2.02 (SD 1.59) by those with normal cognition, 0.93 (SD 1.17) by those with CIWD, and 0.51 (SD 0.86) by those with dementia (Figure 1).

**Table 1:** Descriptive analyses.

**Variable**	***n***	**%**
**Cognition**		
Normal	19.004	80.2
Cognitive Impairment Without Dementia (CIWD)	2.109	8.9
Dementia	2.581	10.8
**Residence Area**		
Urban	17.189	72.5
Rural	6.505	27.4
**Country Region**		
Atlantic	6.202	26.1
Oriental	3.583	15.1
Orinoquia and Amazonia	1.394	5.8
Bogotá	2.003	8.45
Central	6.351	26.8
Pacific	4.161	17.5
**Sex**		
Men	10.112	42.6
Women	13.582	57.3
**Marriage Status**		
Married/With a Partner	12.557	53
Separated/Widower	8.456	35.7
Single	2.671	11.2
**Living Alone**		
Yes	2.201	9.2
No	21.493	90.7
**Health Affiliation**		
Subsidized	14.160	59.82
Contribute	8.998	38.01
No affiliation	512	2.16
**Skin Color**		
Light	11.465	48.3
Medium	8.706	36.7
Dark	3.523	14.8
**Pension**		
Yes	1.589	6.7
No	21.97	93.2
**Lifetime Occupation**		
Manual or dependent worker	15.061	64.89
Boss or independent worker	5.131	22.11
None	3.018	13
**Income**		
<1 minimum wage	13.468	68.7
Between 1 and 2 minimum wage	5074	25.8
More than 2 minimum wage	1.061	5.4
**Physical Capital Tercile**		
1	10.062	42.5
2	7.482	31.6
3	6.15	25.9
**Educational Attainment**		
Less than primary school	14.778	62.6
Completed primary school	6.325	26.8
More than high school	2.498	10.5
**Literacy (Writing)**		
No	18.523	78.2
Yes	5.149	21.7
**Literacy (Reading)**		
No	18.264	77.1
Yes	5.407	22.8

Unadjusted OR estimation showed that people who had an educational qualification lower than the completion of primary school were more likely to develop CIWD (OR 10.18; CI 7.33–14.15) and dementia (OR 8.54; CI 6.52–11.17) than those who had sought further educational qualifications after high school. Reading and writing illiteracy were associated with a higher likelihood of having either CIWD (OR 4.70, CI 4.28–5.17; OR 4.57, CI 4.16–5.02, respectively), or dementia (OR 6.43, CI 5.90–7.02; OR 6.48, CI 5.95–7.07, respectively). Participation in leisure activities was a protective factor against CIWD and dementia (OR 0.56, CI 0.54–0.59; OR 0.36, CI 0.34–0.38, respectively) (Table 2).

**Table 2:** Non-adjusted odds ratio for independent variables.

**Variable**	**Cognitive Impairment**	**Dementia**
	**OR (Confidence Interval)**	**OR (Confidence Interval)**
**Educational Attainment**		
Less than primary school	10.18 (7.33–14.15)^***^	8.54 (6.52–11.17)^***^
Completed primary school	3.95 (2.80–5.56)^***^	2.42 (1.81–3.22)^***^
More than high school	REF	REF
Literacy (Writing)	4.57 (4.16–5.02)^***^	6.48 (5.95–7.07)^***^
Literacy (Reading)	4.70 (4.28–5.17)^***^	6.43 (5.90–7.02)^***^
Leisure Activities	0.56 (0.54–0.59)^***^	0.36 (0.34–0.38)^***^

* *p < 0.05*,

** *p < 0.01*,

*** *p < 0.001*.

*Statistical Significance*.

Analysis of multicollinearity showed high correlation between reading and writing literacy, the former was removed from the multinomial regression models. Table 3 shows the results for sex-stratified multinomial regression models adjusted for potential confounders. In model 1, those who had an educational qualification lower than the completion of primary school (OR 7.27, CI 4.19–12.61) as well as those who had completed primary school (OR 3.02, CI 1.73–5.30) were significantly more likely to have CIWD than their counterparts with higher educational qualifications. Similar results were found for the association between those who had an educational qualification lower than the completion of primary school (OR 3.30, CI 2.06–5.28) and dementia in comparison with those who had the highest level of education. Model 2 for CIWD persisted with the association for those who had an educational qualification lower than the completion of primary school (OR 3.70, CI 2.12–6.47) and those who had completed primary school (OR 2.65, CI 1.51–4.64). Leisure activities were associated with a decreased risk of CIWD (OR 0.73, CI 0.68–0.78). For dementia, in model 2, the effect of educational attainment disappeared, and inability to read was significantly associated with greater risk (OR 2.77, CI 2.29–3.34). Participation in leisure activities decreased the risk of dementia (OR 0.52, CI 0.48– 0.58).

**Table 3:** Multinomial sex-stratified regression models.

	**Women**				**Men**			
**Variable**	**Cognitive impairment without dementia**		**Dementia**		**Cognitive impairment without dementia**		**Dementia**	
	**Model 1^#^**	**Model 2^#^**	**Model 1^#^**	**Model 2^#^**	**Model 1^#^**	**Model 2^#^**	**Model 1^#^**	**Model 2^#^**
	**OR CI 95%**	**OR CI 95%**	**OR CI 95%**	**OR CI 95%**	**OR CI 95%**	**OR CI 95%**	**OR CI 95%**	**OR CI 95%**
**Educational Attainment**								
Less than primary school	7.27^***^ (4.19–12.61)	3.70^***^ (2.12–6.47)	3.30^***^ (2.06–5.28)	1.23 (0.74–2.03)	4.57^***^ (2.71–7.70)	2.42^**^ (1.41–4.14)	3.46^***^ (1.97–6.07)	1.53 (0.83–2.83)
Completed primary school	3.02^***^ (1.73–5.30)	2.65^**^ (1.51–4.64)	1.15 (0.71–1.87)	0.98 (0.59–1.64)	2.47^**^ (1.45–4.21)	2.20^**^ (1.28–3.77)	1.52 (0.84–2.73)	1.31 (0.70–2.45)
More than high school	REF	REF	REF	REF	REF	REF	REF	REF
**Literacy (Reading)**								
No		2.28^***^ (1.90–2.73)		2.77^**^ (2.29 −3.34)		1.94^***^ (1.57–2.41)		1.68^***^ (1.34–2.11)
Yes		REF		REF		REF		REF
**Leisure Activities**		0.73^***^ (0.68–0.78)		0.52^***^ (0.48–0.58)		0.72^***^ (0.66–0.78)		0.48^***^ (0.43–0.53)

# *Adjusted for age, area, marital status, living partners, health affiliation, displacement, country region, race, occupation, salary, and physical assets*.

* *p < 0.05*,

** *p < 0.01*,

*** *p < 0.001*.

*CI, Confidence Interval*.

In adjusted models for men, model 1 for CIWD showed significant results for those who had an educational qualification lower than the completion of primary school (OR 4.57, CI 2.71–7.70) and those who had completed primary school (OR 2.47, CI 1.45–4.21). For dementia, results were significant for those who had an educational qualification lower than the completion of primary school (OR 3.46, CI 1.97–6.07). Model 2 for CIWD persisted with the association for those who had an educational qualification lower than the completion of primary school (OR 2.42, CI 1.41–4.14) and those who had completed primary school (OR 2.20, CI 1.28–3.77) and was significant for the inability to read (OR 1.94, CI 1.57–2.41). Leisure activities were associated with a decreased risk of CIWD (OR 0.72, CI 0.66–0.78). For dementia, in model 2, the effect of educational attainment disappeared; results for reading literacy were significant (OR 1.68, CI 1.34–2.11). Leisure activities decreased the risk of dementia (OR 0.48, CI 0.43–0.53).

## Discussion

This study's main objective was to investigate the effect of participation in leisure activities on reducing the risk of developing CIWD or dementia in Colombian older adults. We found that for CIWD, participation in leisure activities is linked with educational attainment as well as the ability to read. For dementia, participation in leisure activities in later life might impart a protective effect independent of educational attainment for men and women.

In this sample, more than two-thirds of the participants had low educational attainment. Low educational attainment has been associated with a higher risk of dementia and CIWD (33, 34). Our results suggest that when adjusted for leisure activities, the effect of education is significant for CIWD but not for dementia. Education attainment is an essential factor for cognitive reserve building (35); nonetheless, cognitive reserve is not only determined by education but also by other lifestyle factors in which leisure activities are included (4, 36). In individuals with CIWD, educational attainment has been shown to affect intellectual development during the entire adult life span. However, it was not associated with the cognitive decline rate, in contrast with mid/late-life cognitive activities, which was beneficial and delayed the onset of dementia (37). Previous studies had shown the protective effect of cognitive reserve factors such as education and literacy in LMIC (38). Our study contributes with evidence from this region on the potential role of leisure activities on cognitive reserve building, but further research is needed to clarify this association.

Illiteracy was associated as an independent risk factor in non-adjusted analysis for both CIWD and dementia in previous findings (39). Our study included illiteracy while evaluating writing and reading abilities separately; even though the effect of this difference is not known, it is presumed that acquiring one aspect of literacy over the other could lead to differential dementia risks (39). The ability to read provides the means to acquire and structure new knowledge for language skills and reinforces working memory, visual memory, visuospatial processing, and visuomotor skills (40–42). Our results indicate that an inability to read was associated with a higher risk of dementia independent of educational attainment for women; contradictory results have been found in the literature (39, 43).

The study findings suggest that greater participation in leisure activities in later life may be a protective factor against CIWD and dementia among older adults in Colombia after adjusting for education attainment, illiteracy, and other socioeconomic factors covariates. Previous studies have shown the protective effect of participation in leisure activities with a reduced risk of CIWD and dementia (9) and that the relationship between leisure activities and cognition is not driven by educational attainment (44) or SES (45). In contrast to these findings, a recent study based on longitudinal follow-up failed to support that leisure activity participation can lower the risk of dementia but instead suggested that reduction in activity participation indicates possible prodromal dementia (46).

This study has several limitations. First, the study was based on a yes/no self-report questionnaire and frequency, intensity, or quality of the activities was measured. Thus, it is not possible to elucidate the underlying mechanisms for the protective effect described here (12). A study found that participation in several activities with varying cognitive complexity levels was a better predictor of cognitive impairment than increased activity frequency (47). We need to know the amount and frequency that generates a protective effect to be able to design a suitable intervention. Second, although gender, demographic, and SES covariates were adjusted, other known risk factors for dementia such as hypertension, diabetes, stroke, and depression were not included (48). In a cohort of Swedish older adults, moderate to high engagement levels in mental, social, and physical leisure activities were associated with a dramatically decreased risk of dementia in people with diabetes (49). Third, education was measured by years of school completed, and there was no measure of the quality of education. Higher literacy may be a more sensitive marker of cognitive reserve than higher education (50); thus we cannot rule out the possibility that leisure activities' protective effect is dependent on education. Further research is needed to clarify the association between education, leisure activities, and cognitive impairment. Furthermore, data from this study comes from a cross-sectional survey; long term, population-based or representative cohort studies are needed to estimate with more precision the role of lifestyle choices like leisure activities might play in reducing dementia risk (51).

Despite these limitations, this is the first study that demonstrates the protective effect of leisure activities using a survey representative of the Colombian population to the best of our knowledge. The public health implications from this finding are related to the importance of increasing late-life participation in leisure activities, their potential role in late-life cognition, and its benefits in people with and without cognitive impairment (52). Nevertheless, further research is needed to determine the amount of exposure, intervention period, and frequency for designing an effective intervention (53). The available research evidence suggests that it is not too late to increase physical and cognitive activity in old age (14) and policymaking for dementia primary prevention needs evidence for non-pharmacological interventions aiming to increase cognitive reserve (54). More research is required to stablish more reliable conclusions for dementia preventive factors and potential interventions.

## Data Availability Statement

The datasets presented in this article are not readily available because the user agreement does not permit sharing the data directly. Requests to access the datasets should be directed to the Colombian Ministry of Health (repositorio@minsalud.gov.co).

## Ethics Statement

This study was a secondary data analysis approved by the Institutional Review Board of Universidad de los Andes, code ID 1114/2019. And it was consider an investigation without risk according to Colombian Ministry of Health laws.

## Author Contributions

AG and DL were responsible for the study design and data analyses. AG was responsible for drafting the manuscript and interpretation of the findings. DL and BL provided critical feedback on drafts, and approved the final manuscript. All authors contributed to the article and approved the submitted version.

## Conflict of Interest

The authors declare that the research was conducted in the absence of any commercial or financial relationships that could be construed as a potential conflict of interest.

## References

[1] Alzheimer's Disease International. From Plan to Impact: Progress Towards Targets of the Global Action Plan on Dementia. London: Alzheimer's Disease International (2018).

[2] Alzheimer's Disease International. World Alzheimer Report 2015- The Global Impact of Dementia: An Analysis of Prevalence, Incidence, Cost and Trends. London: Alzheimer's Disease International (2015).

[3] Organizacion Panamericana de la Salud ADI. Demencia: Una Prioridad de Salud Publica. Washington: Organizacion Panamericana de la Salud (2013).

[4] Livingston G Huntley J Sommerlad A Ames D Ballard C Banerjee S. Dementia prevention, intervention, and care: 2020 report of the Lancet Commission. Lancet. (2020) 396:413–46. 10.1016/S0140-6736(20)30367-6 32738937 PMC7392084

[5] Fallahpour M Borell L Luborsky M Nygård L. Leisure-activity participation to prevent later-life cognitive decline: a systematic review. Scand J Occup Ther. (2015) 23:162–97. 10.3109/11038128.2015.1102320 26586025

[6] Karp A Paillard-Borg S Wang HX Silverstein M Winblad B Fratiglioni L. Mental, physical and social components in leisure activities equally contribute to decrease dementia risk. Dement Geriatr Cogn Disord. (2006) 21:65–73. 10.1159/000089919 16319455

[7] Wa Lam LC Cheng ST. Maintaining long-term adherence to lifestyle interventions for cognitive health in late life. Int Psychogeriatr. (2013) 25:171–3. 10.1017/S1041610212001603 23010393

[8] Cheng ST Chow PK Yu ECS Chan ACM. Leisure activities alleviate depressive symptoms in nursing home residents with very mild or mild dementia. Am J Geriatr Psychiatry. (2012) 20:904–8. 10.1097/JGP.0b013e3182423988 22377774

[9] Yates LA Ziser S Spector A Orrell M. Cognitive leisure activities and future risk of cognitive impairment and dementia: systematic review and meta-analysis. Int Psychogeriatr. (2016) 28:1791–806. 10.1017/S1041610216001137 27502691

[10] Tolppanen AM Solomon A Kulmala J Kåreholt I Ngandu T Rusanen M. Leisure-time physical activity from mid- to late life, body mass index, and risk of dementia. Alzheimers Dement. (2015) 11:434–43.e6. 10.1016/j.jalz.2014.01.008 24721528

[11] Akbaraly TN Portet F Fustinoni S Dartigues JF Artero S Rouaud O. Leisure activities and the risk of dementia in the elderly: results from the three-city study. Neurology. (2009) 73:854–61. 10.1212/WNL.0b013e3181b7849b 19752452

[12] Sajeev G Weuve J Jackson JW Vanderweele TJ Bennett DA Grodstein F. Late-life cognitive activity and dementia. Epidemiology. (2016) 27:732–42. 10.1097/EDE.0000000000000513 27227783 PMC5460628

[13] Scarmeas N Stern Y. Cognitive reserve and lifestyle. J Clin Exp Neuropsychol. (2003) 25:625–33. 10.1076/jcen.25.5.625.14576 12815500 PMC3024591

[14] Cheng ST. Cognitive reserve and the prevention of dementia: the role of physical and cognitive activities. Curr Psychiatry Rep. (2016) 18:85. 10.1007/s11920-016-0721-2 27481112 PMC4969323

[15] Stern Y. Cognitive reserve and Alzheimer disease. Alzheimer Dis Assoc Disord. (2006) 20:112–7. 10.1097/01.wad.0000213815.20177.19 16772747

[16] Scarmeas N Levy G Tang M-X Manly J Stern Y. Influence of leisure activity on the incidence of Alzheimer's Disease. Neurology. (2001) 57:2236–42. 10.1212/WNL.57.12.2236 11756603 PMC3025284

[17] Crowe M Andel R Pedersen NL Johansson B Gatz M. Does participation in leisure activities lead to reduced risk of Alzheimer's disease? A prospective study of Swedish twins. J Gerontol Ser B Psychol Sci Soc Sci. (2003) 58:249–55. 10.1093/geronb/58.5.P249 14507930

[18] Richards M Hardy R Wadsworth MEJ. Does active leisure protect cognition? Evidence from a national birth cohort. Soc Sci Med. (2003) 56:785–92. 10.1016/S0277-9536(02)00075-8 12560011

[19] Cerin E Leslie E. How socio-economic status contributes to participation in leisure-time physical activity. Soc Sci Med. (2008) 66:2596–609. 10.1016/j.socscimed.2008.02.012 18359137

[20] Cassarino M Setti A. Environment as ‘Brain Training': a review of geographical and physical environmental influences on cognitive ageing. Ageing Res Rev. (2015) 23:167–82. 10.1016/j.arr.2015.06.003 26144974

[21] Gonzalez FJ Gaona C Quintero M Chavez CA Selga J Maestre GE. Building capacity for dementia care in Latin America and the Caribbean Francisco. Dement e Neuropsychol. (2014) 8:310–6. 10.1590/S1980-57642014DN84000002 PMC4412169 25932285

[22] Mukadam N Sommerlad A Huntley J Livingston G. Population attributable fractions for risk factors for dementia in low-income and middle-income countries: an analysis using cross-sectional survey data. Lancet Glob Heal. (2019) 7:e596–603. 10.1016/S2214-109X(19)30074-9 31000129 PMC7617123

[23] Custodio N Wheelock A Thumala D Slachevsky A. Dementia in Latin America: epidemiological evidence and implications for public policy. Front Aging Neurosci. (2017) 9:221. 10.3389/fnagi.2017.00221 28751861 PMC5508025

[24] Baez S Ibáñez A. Dementia in Latin America: an emergent silent tsunami. Front Aging Neurosci. (2016) 8:253. 10.3389/fnagi.2016.00253 27840605 PMC5083841

[25] Parra MA Baez S Allegri R Nitrini R Lopera F Slachevsky A. Dementia in Latin America Assessing the present and envisioning the future. Neurology. (2018) 90:222–31. 10.1212/WNL.0000000000004897 29305437 PMC5791795

[26] Gomez F Corchuelo J Curcio CL Calzada MT Mendez F. SABE Colombia: survey on health, well-being, and aging in Colombia - study design and protocol. Curr Gerontol Geriatr Res. (2016) 2016:7910205. 10.1155/2016/7910205 27956896 PMC5124445

[27] Donovan GO Hamer M Sarmiento OL Hessel P. Education in early life markedly reduces the probability of cognitive impairment in later life in Colombia. Sci Rep. (2020) 10:17685. 10.1038/s41598-020-74822-2 33077810 PMC7572407

[28] Garcia-Cifuentes E Márquez I Vasquez D Aguillon D Borda MG Lopera F. The role of gait speed in dementia: a secondary analysis from the SABE Colombia study. Dement Geriatr Cogn Disord. (2020) 1–8. 10.1159/000510494 33207340

[29] Pérez-Sousa MÁ del Pozo-Cruz J Olivares PR Cano-Gutiérrez CA Izquierdo M Ramírez-Vélez R. Role for physical fitness in the association between age and cognitive function in older adults: a mediation analysis of the SABE Colombia study. Int J Environ Res Public Health. (2021) 18:751. 10.3390/ijerph18020751 33477293 PMC7829928

[30] Icaza MG Albala C. Minimental State Examinations (MMSE) del Estudio de Demencia en Chile: Analisis Estadistico. Washington, DC: OPS Investig en Salud Pública Doc Técnicos. (1999) p. 7.

[31] Luck T Luppa M Wiese B Maier W Den Bussche H Van Eisele M. Prediction of incident dementia: Impact of impairment in instrumental activities of daily living and mild cognitive impairment-results from the german study on ageing, cognition, and dementia in primary care patients. Am J Geriatr Psychiatry. (2012) 20:943–54. 10.1097/JGP.0b013e31825c09bc 22706332

[32] Ministerio de Salud y Protección Social. Oficina de Protección Social-MINSALUD, Departamento Administrativo de ciencia tecnología e innovación-C-. SABE Colombia 2015: Estudio Nacional de Salud, Bienestar y Envejecimiento. Bogotá: Minsalud. (2015).

[33] Caamaño-Isorna F Corral M Montes-Martínez A Takkouche B. Education and dementia: a meta-analytic study. Neuroepidemiology. (2006) 26:226–32. 10.1159/000093378 16707907

[34] Sharp ES Gatz M. Relationship between education and dementia an updated systematic review. Alzheimer Dis Assoc Disord. (2011) 25:289–304. 10.1097/WAD.0b013e318211c83c 21750453 PMC3193875

[35] Whalley LJ Deary IJ Appleton CL Starr JM. Cognitive reserve and the neurobiology of cognitive aging. Ageing Res Rev. (2004) 3:369–82. 10.1016/j.arr.2004.05.001 15541707

[36] Polidori MC Nelles G Pientka L. Prevention of dementia: focus on lifestyle. Int J Alzheimers Dis. (2010) 2010:393579. 10.4061/2010/393579 20721289 PMC2915647

[37] Vemuri P Lesnick TG Przybelski SA Machulda M Knopman DS Mielke MM. Association of lifetime intellectual enrichment with cognitive decline in the older population. JAMA Neurol. (2014) 71:1017–24. 10.1001/jamaneurol.2014.963 25054282 PMC4266551

[38] Prince M Acosta D Ferri CP Guerra M Huang Y Rodriguez JJL. Dementia incidence and mortality in middle-income countries, and associations with indicators of cognitive reserve: a 10/66 Dementia Research Group population-based cohort study. Lancet. (2012) 380:50–8. 10.1016/S0140-6736(12)60399-7 22626851 PMC3525981

[39] Arce Rentería M Vonk JMJ Felix G Avila JF Zahodne LB Dalchand E. Illiteracy, dementia risk, and cognitive trajectories among older adults with low education. Neurology. (2019) 93:E2247–56. 10.1212/WNL.0000000000008587 31722961 PMC6937498

[40] Bramão I Mendonça A Faísca L Ingvar M Peterson KM Reis A. The impact of reading and writing skills on a visuo-motor integration task: a comparison between illiterate and literate subjects. J Int Neuropsychol Soc. (2007) 13:359–64. 10.1017/S1355617707070440 17286893

[41] Petersson KM Reis A Ingvar M. Cognitive processing in literate and illiterate subjects: a review of some recent behavioral and functional neuroimaging data. Scand J Psychol. (2001) 42:251–67. 10.1111/1467-9450.00235 11501739

[42] Kosmidis MH Zafiri M Politimou N. Literacy versus formal schooling: influence. Arch Clin Neuropsychol. (2011) 26:575–82. 10.1093/arclin/acr063 21810857

[43] Zhang M Katzman R Salmon D Jin H Cai G Wang Z. The prevalence of dementia and Alzheimer's disease in Shanghai, China: impact of age, gender, and education. Ann Neurol. (1990) 27:428–37. 10.1002/ana.410270412 2353798

[44] Peterson RL Gilsanz P George KM Ackley S Glymour MM Mungas DM. Differences in association of leisure time activities and cognition in a racially/ethnically diverse cohort of older adults: findings from the KHANDLE study. Alzheimers Dement Transl Res Clin Interv. (2020) 6:1–9. 10.1002/trc2.12047 32607410 PMC7317643

[45] Singh-Manoux A Richards M Marmot M. Leisure activities and cognitive function in middle age: Evidence from the Whitehall II study. J Epidemiol Community Health. (2003) 57:907–13. 10.1136/jech.57.11.907 14600119 PMC1732329

[46] Sommerlad A Sabia S Livingston G Kivimäki M Lewis G Singh-Manoux A. Leisure activity participation and risk of dementia: 18 year follow-up of the Whitehall II Study. Neurology. (2020) 95:e2803–15. 10.1212/WNL.0000000000010966 33115773 PMC7734721

[47] Carlson MC Parisi JM Xia J Xue QL Rebok GW Bandeen-Roche K. Lifestyle activities and memory: variety may be the spice of life the women's health and aging study II. J Int Neuropsychol Soc. (2012) 18:286–94. 10.1017/S135561771100169X 22172155 PMC3508669

[48] Frankish H Horton R. Prevention and management of dementia: a priority for public health. Lancet. (2017) 390:2614–5. 10.1016/S0140-6736(17)31756-7 28735854

[49] Marseglia A Wang HX Rizzuto D Fratiglioni L Xu W. Participating in mental, social, and physical leisure activities and having a rich social network reduce the incidence of diabetes-related dementia in a cohort of Swedish older adults. Diabetes Care. (2019) 42:232–9. 10.2337/dc18-1428 30523030

[50] Kaup AR Simonsick EM Harris TB Satterfield S Metti AL Ayonayon HN. Older adults with limited literacy are at increased risk for likely dementia. J Gerontol Ser A Biol Sci Med Sci. (2014) 69:900–6. 10.1093/gerona/glt176 24158765 PMC4067115

[51] Henderson VW Elias MF. Leisure activity for dementia prevention: more work to be done. Neurology. (2020) 95:895–6. 10.1212/WNL.0000000000010962 33115774

[52] Wells RE Kerr C Dossett ML Danhauer SC Sohl SJ Sachs BC. Can adults with mild cognitive impairment build cognitive reserve and learn mindfulness meditation? Qualitative theme analyses from a small pilot study. J Alzheimers Dis. (2019) 70:825–42. 10.3233/JAD-190191 31282418 PMC6753379

[53] Iizuka A Suzuki H Ogawa S Kobayashi-Cuya KE Kobayashi M Takebayashi T. Can cognitive leisure activity prevent cognitive decline in older adults? A systematic review of intervention studies. Geriatr Gerontol Int. (2019) 19:469–82. 10.1111/ggi.13671 31020777

[54] Wu YT Fratiglioni L Matthews FE Lobo A Breteler MMB Skoog I. Dementia in western Europe: epidemiological evidence and implications for policy making. Lancet Neurol. (2016) 15:116–24. 10.1016/S1474-4422(15)00092-7 26300044

